# Urological impact of race-free estimated glomerular filtration rate equations

**DOI:** 10.1590/S1677-5538.IBJU.2023.9913

**Published:** 2024-02-07

**Affiliations:** 1 Universidade Estadual de Campinas Campinas SP Brasil UroScience, Universidade Estadual de Campinas (Unicamp). Campinas, SP, Brasil;; 2 Harvard Medical School Brigham and Women's Hospital Division of Urological Surgery Boston MA USA Division of Urological Surgery, Brigham and Women's Hospital, Harvard Medical School. Boston, MA, USA;; 3 Universidad Austral Hospital Universitario Austral Provincia de Buenos Aires Argentina Servicio de Urología, Hospital Universitario Austral, Universidad Austral. Provincia de Buenos Aires, Argentina;; 4 Dana-Farber Cancer Institute Lank Center for Genitourinary Oncology Boston MA USA Lank Center for Genitourinary Oncology, Dana-Farber Cancer Institute. Boston, MA, USA;; 5 Pontifícia Universidade Católica de Campinas Faculdade de Ciências da Vida Departamento de Oncologia Urológica Campinas SP Brasil Departamento de Oncologia Urológica, Faculdade de Ciências da Vida, Pontifícia Universidade Católica de Campinas (PUC-Campinas). Campinas, SP, Brasil

## COMMENT

ForHistorically, estimated glomerular filtration rate (eGFR) have used equations that include a correction factor for race. This correction factor was introduced based on observed differences in serum creatinine levels between individuals of different races, with higher levels generally seen in people of African ancestry. However, in recent years there has been growing concern that the use of race as a factor in GFR calculations may not accurately reflect an individual's kidney function since we typically only use "Black" or "Non-black" as their racial categories and we know that this simplification does not reflect the real ethnic diversity of the global population (1).

In response to this context, the National Kidney Foundation and American Society of Nephrology Task Force recommended "race-free" eGFR calculations, and the two main formulas used to calculate the glomerular filtration rate – Chronic Kidney Disease Epidemiology Collaboration (CKD-EPI) and Modification of Diet in Renal Disease (MDRD) – have been modified. The last CKD-EPI equation, adopted in 2021, does not include an adjustment for race and the MDRD has made the criterion "race" optional in calculating GFR. These changes carry substantial implications for Black African ancestry patients (2).

Although the European Federation of Clinical Chemistry and Laboratory Medicine (EFLM) opposes such a change in the calculation, the current race coefficient has been shown to be inapplicable to Black populations outside the USA, like in Europe, Africa, and Brazil. In these areas, the CKD-EPI equation without the race coefficient demonstrated better performance than the MDRD equation with the race coefficient (3, 4).

Most research studies to date that have evaluated the consequence of removing the race adjustment form the eGFR calculation formulas are simulations of alternative approaches that estimate the number of Black adults potentially affected by shifting eGFR across thresholds commonly used for clinical decision making. For CKD screening, it could increase the number of Black adults meeting a threshold by 16% to >100% (local vs national studies) and the number of Black adults meeting eGFR <30 mL/min/1.73m2 by up to 52%, with potential benefit or harm resulting from an increased diagnosis or overdiagnosis, respectively. It could also increase by 29%-52% the number of Black adults being eligible for Medicare's medical nutrition or kidney disease education benefit, vascular access referral, or transplant referral (5-7).

For patients with cancer, it would impact the cross-sectional imaging in diagnosing or staging and the type of urinary diversion following cystectomy, increasing the number of Black individuals ineligible for chemotherapy or those recommended to receive less than a full dose or a potentially less accurate dose adjustment (by 61%-163%) for several chemotherapies, which may lead to decreased quality of life and survival (8), thereby worsening the already prevalent racial disparities in healthcare. Furthermore, this might intensify the ongoing issue of underrepresentation by potentially increasing the exclusion of Black patients from clinical trials (9).

Another implication might be related to the disparity on potential kidney donors and qualification for renal transplantation, differences between race-based and race-free estimates might lead to disparate exclusion of donor candidates, increasing the exclusion of Black donor candidates (10).

Race-free eGFR equations have higher bias and lower agreement between measured and estimated GFR categories, compared to their predecessors. While the process for reporting race is not entirely clear, race can represent a genetically and socially heterogeneous group and race in eGFR equations bring a social and a biologic construct where race coefficients among the global ancestry proportions determines the genetic ancestry in percentage derived from genome-scale genetic data (11).

Considering the impact of the new guidelines on clinical decision making, we expect to see more data comparing old and new approaches. Is it time to review the treatment of patients who fit the old criteria? Or should we insist on adjusting GFR equations by exploring more ethnic diversity? Whatever the opinion, randomized trials are needed to compare patients who have changed classification with the new GFR equations and understand how they perform.
